# Toxicity of representative mixture of five rare earth elements in juvenile rainbow trout (*Oncorhynchus mykiss)* juveniles

**DOI:** 10.1007/s11356-020-12218-5

**Published:** 2021-02-03

**Authors:** Houda Hanana, Christine Kleinert, François Gagné

**Affiliations:** Aquatic Contaminants Research Division, Environnement and Climate Change Canada, 105 McGill, Montréal, QC H2Y 2E7 Canada

**Keywords:** Rare earth elements, Mixtures, Oxidative stress, Toxicity

## Abstract

Rare earth elements (REEs) are contaminants of increasing interest due to intense mining activities for commercial purposes and ultimately released in the environment. We exposed juvenile rainbow trout (*Oncorhynchus mykiss*) to a representative mixture of the five most abundant REEs for 96 h at concentrations similar found in lakes contaminated by mining activities at 0.1, 1, 10, and 100X whereas the 1x mixture contained cerium (Ce, 280 μg/L), lanthanum (La, 140 μg/L), neodymium (Nd, 120 μg/L), praseodymium (Pr, 28 μg/L), and samarium (Sm, 23 μg/L). We investigated the expression of 14 genes involved in oxidative stress, DNA repair, tissue growth/proliferation, protein chaperoning, xenobiotic biotransformation, and ammonia metabolism in the liver. In addition, DNA damage, oxidative stress (lipid peroxidation or LPO), inflammation (cyclooxygenase or COX activity), detoxification mechanisms (glutathione-S-transferase activity or GST), and labile zinc were determined in gills. The data revealed that genes involved in oxidative stress-catalase (*cat*), heat shock proteins 70 (*hsp70*), and glutamate dehydrogenase (*glud*) were upregulated while glutathione S-transferase (*gst*) and metallothionein (*mt*) gene expressions were downregulated. The mixture was genotoxic and increased labile Zn in gills of exposed trout. These changes occurred at concentrations 600 times lower than the LC_50_ for this mixture indicating effects below the 1X concentration. Based on principal component analysis and concentration-dependent reponses, the following sublethal effects were considered the most important/significant: DNA strand breaks (genotoxicity), labile Zn, *cat*, *gst*, *hsp70*, *sparc*, *mt*, *and glud.* These effects of fish juveniles are likely to occur in environments under the influence of mining activities.

## Introduction

Rare earth elements (REEs) comprise the 15 elements of the lanthanide family in addition to scandium and yttrium. Demand for these chemicals is increasing, as they are essential for a number of technological products including smartphones and miscellaneous computer parts. As recycling rates of REEs are below 1% (Binnemans et al. 2013), mining efforts have to be increased to meet demand and suggests that REEs could find their way in the environment.

REEs are considered contaminants of emerging interest, as mining efforts and subsequent release into the aquatic environment raise concerns about the ecotoxicological impacts to the aquatic environment. This group of chemicals has already been detected in the marine (Moermond et al. 2001), freshwater (Kulaksiz and Bau 2011), and soil environment (Tranchida et al. 2011) as well as biota (Tu et al. 1994; Bustamante and Miramand 2005; Hu et al. 2006; Censi et al. 2013; Fu et al. 2014; Mayfield and Fairbrother 2015). However, information on potential adverse toxicological effects and toxicity mechanisms is still scarce to date for this group of elements.

The toxicological investigations of a number of REEs have been studied in fish and aquatic invertebrates. Lanthanum (La^3+^) and Cerium (Ce^3+^) are the most frequently studied REEs owing to their abundance and use. Zebra fish (*Danio rerio*) embryo and larval development were delayed; survival and hatching rates were decreased and tail malformations occurred after La exposure (Cui et al. 2012). Lanthanum chloride (LaCl_3_) has been shown to affect acetylcholinesterase kinetics in electric eel (*Electrophorus electricus*) (Tomlinson et al. 1982) as well as electroreceptor sensitivity in catfish (*Kryptopterus sp.*) (Roth 1982). Furthermore, growth arrest DNA repair gene (*gadd45*) expression was increased 2-fold relative to controls after 96 h exposure to La in juvenile rainbow trout (*Oncorhynchus mykiss*) (Dubé et al. 2019). This acute lethality assay in juvenile rainbow trout is a standardized bioassay and currently used to regulate the toxicity of wastewaters under the Canadian Environmental Protection Act. In aquatic invertebrates, a variety of biochemical processes have been shown to be affected by La exposure including but not limited to lipid peroxidation (LPO), gluthathione-S-transferase (GST) activity, and citrate synthase (Hanana et al. 2017). Furthermore, La has displayed embryotoxicity in several sea urchin species (Trifuoggi et al. 2017).

Cerium (Ce) was frequently tested in its nanoparticle form. Cerium oxide nanoparticles (nCeO_2_) showed elevated plasma cortisol levels in white sucker (*Catostomus commersonii*) after acute in vivo exposure to 1 mg/L nCeO_2_ for 25 h. Biomarkers for oxidative, cardiorespiratory, or osmoregulatory stress, however, remained unchanged, suggesting mild toxicity effects outside of the cardiorespiratory system (Rundle et al. 2016). Heat shock protein 70 (*hsp70*) gene expression significantly increased after 96 h exposure to Ce^3+^ in juvenile rainbow trout (Dubé et al. 2019) indicating a stress response related to exposure. Furthermore, Ce^3+^ was tested for its antioxidant capacities in the liver of Silver crucian carp (*Carassius gibelio*) injected with lead (Pb). It decreased reactive oxygen species (ROS) production and LPO while improving catalase, superoxide dismutase (SOD), and ascorbate peroxidase activities (Ling and Hong 2010). Ce effects in aquatic invertebrates include morphological changes at relatively low concentrations (EC_50_ = 0.05 mg/L) and mortality (LC_50_ = 0.33 mg/L) in *Hydra attenuata* after 96 h exposure (Blaise et al. 2018). Furthermore, Ce has displayed embryotoxicity in several sea urchin species (Trifuoggi et al. 2017). Neodymium (Nd) nanoparticles (nNd_2_O_3_) affected heart rate, the cerebrovascular system, and neural apoptosis in zebra fish embryos (Chen et al. 2020). Furthermore, Nd exposure significantly increased *hsp70* gene expression in rainbow trout (Dubé et al. 2019). Nd effects in aquatic invertebrates include induced morphological changes (EC_50_ = 0.09 mg/L) and mortality (LC_50_ = 0.31 mg/L) in *Hydra attenuata* after 96 h exposure (Blaise et al. 2018). Moreover, Nd has displayed embryotoxicity in several sea urchin species (Trifuoggi et al. 2017). Samarium (Sm) significantly increased *cyp1a1* gene expression in rainbow trout (Dubé et al. 2019) indicating possible perturbation in phase I biotransformation of non-polar aromatic hydrocarbons. It was the second most toxic compound with Yttrium to juvenile rainbow trout in the above study. In aquatic invertebrates, Sm^3+^ has been shown to affect gene expression of GST, catalase (*cat*), cytochrome c oxidase 1 (*CO1*), and *cyclin D* (Hanana et al. 2018). Furthermore, both prostaglandin cyclooxygenase (COX) activity and DNA strand breaks were decreased (Hanana et al. 2018). In *Hydra attenuata,* Sm induced morphological changes (EC_50_ = 0.18 mg/L) and mortality (LC_50_ = 0.77 mg/L) after 96 h exposure (Blaise et al. 2018). Sm oxide nanoparticles (nSm_2_O_3_) were also considered toxic in another study on *Hydra* (Blaise et al. 2008). Furthermore, Sm has displayed embryotoxicity in several sea urchin species (Trifuoggi et al. 2017). Finally, praseodymium (Pr) effects in aquatic invertebrates include induced morphological changes (EC_50_ = 0.02 mg/L) and mortality (LC_50_ = 0.56 mg/L) in *Hydra attenuata* after 96 h exposure (Blaise et al. 2018).

The majority of the toxicological database comprises two of the elements: cerium (Ce) and lanthanum (La) but virtually no information exists about environmentally realistic mixtures. In our study, we wanted to investigate the effect of an environmentally relevant mixture of the first 5 most-abundant REEs (La, Ce, Nd, Sm, and Pr) based on reported values in lakes contaminated by mining activities in the North of Quebec (Canada) (Beaubien 2015). The first five most abundant REEs were prepared at different concentrations but keeping the same proportion between them and exposed to juvenile rainbow trout for toxicity investigations. Compared to natural lakes, these concentrations are 2–3 orders of magnitudes higher and with similar relative ratios. We evaluated genotoxicity (DNA strand breaks), mRNA expression of a set of genes involved in oxidative stress, Fe3+ /divalent metal homeostatis, DNA repair, protein denaturation, cell growth/proliferation and intermediary metabolism and biochemical markers of oxidative stress (lipid peroxidation), inflammation (cyclooxygenase activity), detoxification mechanisms (glutathione-S-transferase activity), and labile zinc, which is a biomarker for metal exposure, in the gills and liver.

## Material and methods

### Exposure of rainbow trout to a mixture of selected REEs

Rainbow trout juveniles (length: 7.7 cm ± 0.2 cm; weight: 4.2 g ± 0.3 g) were maintained for 2 weeks at 15 °C on a photoperiod of 16 h light/ 8 h dark and constant aeration, following a standardized protocol of Environment and Climate Change (Environmental Protection Series 1990). The aquarium water obtained from UV-treated, charcoal-filtered tap water from the city of Montreal (QC, Canada). Trout were used to determine the lethal and sublethal toxicity of a mixture of 5 REEs (lanthanum, cerium, neodymium, samarium, and praseodymium) after 96 h exposure to environmentally relevant concentrations (1x) and incremental concentrations ranging from 0.1x to 100x (Table 1). The toxicity was also examined with the REEs individually. The nominal concentrations were selected based on the concentrations found in the aquatic environment contaminated by mining activities (Beaubien 2015).

**Table 1:** Concentrations of the individual lanthanides in the REE mixture. The 1x concentration is based on measured concentrations in a lake contaminated by mining activities (Beaubien 2015)

Concentration (μg/L)	Lanthanum La	Cerium Ce	Neodymium Nd	Samarium Sm	Praseodymium Pr
0.1x	14	28	12	2.3	2.8
1x	140	280	120	23	28
10x	1400	2800	1200	230	280
100x	14,000	28,000	12,000	2300	2800

For each lanthanide, stock solutions were prepared one day prior to exposure beginning by dissolving the chloride salts of each REE in distilled water. The final concentrations correspond to the amount of elemental ions in each solution. The stock solutions were further diluted to obtain the 100x REE mixture, which was then used to obtain the 0.1x, 1x, and 10x dilutions. For each concentration,1 L was prepared and added to a final volume of 60 L tap water (dechlorinated, UV-treated tap water from the city of Montreal). Then, the rainbow trout were placed in polyethylene bag-lined 60 L containers (*n* = 13 fish per concentration). The trout in the negative control (NC) was exposed to tap water only. Physico-chemical parameters of the aquarium water were monitored daily. No difference in physico-chemical parameters was observed between different concentrations of the REE exposure mediums. Fish were additionally monitored daily for signs of distress, behavior changes, and mortality.

At the end of the exposure, the 96 h median lethal concentration that led to 50% mortality (LC_50_) was determined with the Spearman-Karber method (Finney 1964) using CETIS software (version 1.8.7.7). Trout were euthanized with 0.1% MS222 (Sigma-Aldrich, Oakville, ON, Canada) according to the recommendations of the animal care committee. Fish exposed to 100x REE mixture were not used for further experiments due to the high mortality observed (61.5%). The length and weight of each fish were recorded. Then, the gills and livers were immediately dissected. Livers were stored in RNA*later* (Thermo Fisher Scientific, Burlington, ON, Canada) at − 20 °C until gene expression analysis (*n* = 9 per concentration). The gills (*n* = 9 per concentration) were stored at − 80 °C prior to homogenization for biomarker assays.

### Biomarker analyses

Gill samples were homogenized at a 1:15 (*W*/*V*) ratio in 25 mM HEPES-NaOH buffer (pH 7.4) containing 140 mM NaCl, 0.1 mM dithiothreitol, and 1 mg/L aprotinin. A subsample of the homogenate was centrifuged at 15,000×*g* for 20 min at 2 °C and the supernatant (S15) was carefully collected to measure labile Zinc levels, cyclooxygenase (COX), and glutathione-S-transferase (GST) activities. The remaining homogenates were used to determinate lipid peroxidation (LPO) and DNA damage (DNA strand breaks with the alkaline precipitation assay). Total protein concentrations were determined in the homogenate and the S15 fraction using standard solutions of albumin for calibration (Bradford 1976) and all samples were stored at − 80 °C after homogenization until further analysis.

DNA damage was assessed with a modified alkaline precipitation assay (Olive 1988; Gagné, 2014). A solution containing 200 μL of 2% SDS, 10 mM Tris, 10 mM EDTA, and 40 mM NaOH was added to 25 μL of gill homogenates and incubated for 1 min. Then, 200 μL of 120 mM KCI was added to the mixture, and samples were incubated at 60 °C for 10 min. The DNA was precipitated by placing the samples on ice for 20 min and then centrifuging at 8000×*g* and 4 °C for 5 min. DNA strand breaks in the supernatant were detected using Hoechst dye (West et al. 1985). Therefore, 50 μL supernatant was carefully removed and mixed with 150 μL buffer containing 400 mM NaCl, 4 mM cholate, 100 mM Tris (pH 8.5), containing 1 μg/mL Hoechst (Thermo Fisher Scientific, Burlington, ON, Canada). Fluorescence was read at 360 nm excitation/ 460 nm emission using the Synergy 4 microplate reader (BioTek, Winooski, VT, USA). A salmon sperm DNA (Sigma-Aldrich, Oakville, ON, Canada) standard curve was used to quantify DNA content in supernatant. The data were expressed as μg DNA/ mg proteins.

Lipid damage was determined by measuring lipid peroxidation (LPO) according to the thiobarbituric acid (TBARS) method (Wills 1987). Accordingly, 150 μL of 20% trichloroacetic acid containing 2 mM FeSO_4_ and 75 μL of 0.67% thiobabituric acid were added to 75 μl gill homogenate. The mixture was incubated at 70 °C for 10 min, cooled to room temperature and 100 μL per sample was transferred to black half-area 96-well microplates. Fluorescence was determined at 540 nm excitation/ 600 nm emission using the Synergy 4 microplate reader (BioTek, Winooski, VT, USA). Blanks and standards of tetramethoxypropane (stabilized form of malonaldehyde) were prepared using homogenization buffer which was used as a standard. The data were expressed as nmol TBARS/ mg proteins.

Cyclooxygenase (COX) activity was determined using a microplate fluorescence procedure. The assay is based on the formation of H_2_O_2_ detected by the oxidation of 2,7-dichlorofluorescein substrate in the presence of arachidonate and horseradish peroxidase (Fujimoto et al. 2002). Briefly, 25 μL of the gill S15 fraction was mixed with 150 μL of assay buffer consisting of 50 mM Tris-Acetate, 0.5 mM EDTA, and 0.1% Tween 20 (pH 8.0). Then, 0.12 mM arachidonate, 0.1 mM dichlorofluorescein diactetate, and 0.1 μg/mL horseradish peroxidase in 50 mM KH_2_PO_4_ (pH 8.0) are added. The reaction mixture was incubated for a total of 30 min at 25 °C, and the formation of fluorescein was measured every 2 min at 485 nm excitation and 520 nm emission using the Synergy 4 microplate reader (BioTek, Winooski, VT, USA). The data were expressed as relative fluorescein units/min/mg proteins.

Glutathione-S-transferase (GST) activity was determined using 1-chloro-2,4-dinitrobenzene (CDNB) as substrate (Gagne 2014). Briefly, 30 μL S15 fraction was mixed with 200 μL 1 mM reduced l-glutathione (GSH), 1 mM 1-chloro-2,4-dinitrobenzene (CDNB), and 125 mM NaCl in 10 mM HEPES (pH 6.5). Absorbance was read in clear microplates at 340 nm every 1 min for 30 min using the Synergy 4 microplate reader (BioTek, Winooski, VT, USA). The data were expressed as GSH (nmol) / min/ mg proteins.

Labile Zn levels in tissues were determined using the fluorescent probe N-(6-Methoxy-8-Quinolyl)-p-Toluenesulfonamide (TSQ) (Gagné and Blaise 1996). Accordingly, 20 μL of the gill S15 fraction was combined with 80 μL of 100 μM TSQ, prepared in 20% DMSO in 5 mM KH_2_PO_4_ (pH 7.4) and 140 mM NaCl. The mixture was agitated for 5 min and fluorescence was determined in black 96-well half-area plates at 360 nm excitation/ 460 nm emission using the Synergy 4 microplate reader (BioTek, Winooski, VT, USA). Data were assessed using a zinc chloride (Sigma-Aldrich, Oakville, ON, Canada) standard curve and expressed as ng Zn/ mg proteins.

### RNA extraction and reverse transcription

Total RNA was extracted with the RNA Plus Mini kit and QIAShredder columns (QIAGEN, Toronto, ON, Canada) following the manufacturer’s instructions. RNA was eluted in 30 μL RNase-free water. RNA concentration and purity were measured with the NanoDrop 1000 (Thermo Fisher Scientific, Burlington, ON, Canada). A260/A280 ratios of samples were between 1.9–2.16. RNA concentration was adjusted to 40 ng/μL for all samples.

Total RNA was reverse transcribed into cDNA using the Quanti-Tect® reverse transcription kit (QIAGEN, Toronto, ON, Canada) following the manufacturer’s instructions. Briefly, 100 ng RNA (2.5 μL) was added to 2 μL 7x genomic DNA wipe-out buffer and 9.5 μL RNase-free water for a total volume of 14 μL in thin-walled PCR tubes (Bio-Rad, Mississauga, ON, Canada). To eliminate genomic DNA, samples were placed on a thermocycler at 42 °C for 5 min. Tubes were placed on ice and 4 μL Quantiscript RT buffer, 1 μL RT Primer Mix, and 1 μL Quantiscript reverse transcriptase was added to the gDNA elimination reaction. The RT reaction was incubated at 42 °C for 15 min, and then at 95 °C for 3 min to inactivate the reverse transcriptase. All samples were diluted (1:10) with DEPC-H_2_O and stored at − 80 °C before real-time quantitative PCR analysis (qPCR).

### Real-time qPCR

Table 2 shows the selected genes for this study and their respective primers. Several primers have been previously published. We designed additional primer pairs using Primer-BLAST from NCBI (Primer3 with Blast; Rozen and Skaletsky, 2000). We assessed the absence of secondary structures using Netprimer (Biosoft, PaloAlto, CA, USA). We evaluated at least two primer pairs for each gene. All primers were synthetized by IDT, Integrated DNA Technologies (Coralville, IA, USA).

**Table 2:** List of target and reference genes

Genes of interest	Symbol	Function	Forward-primer 5′-3’ Reverse-primer 5′-3′	Amplicon (bp)	Reference
Catalase	CAT	Removal of H2O2	F-TGATGTCACACAGGTGCGTA R-GTGGGCTCAGTGTTGTTGAG	195	(Fontagne et al. 2008)
Cytochrome C oxidase 1	CO1	Complex 4 of the respiratory chain	F-ATCCTGTCGTTTGTGTTGGC R-GGTTGTGGAAGAGAGAGTGGTT	172	In house
Cytochrome P450 1A	CYP1A	Hydroxylation of polaromatic hydrocarbons	F-GATGTCAGTGGCAGCTTTGA R-TCCTGGTCATCATGGCTGTA	104	(Aluru and Vijayan, 2006)
Ferritin H	FeH	Fe^3+^ homeostatis	F-TGGGCAAAGAGAGCACATAGA R-GGAACGGGAGGGAATAGCAT	143	In house
Glutamate dehydrogenase	GLUD	Assimilation of ammonia	F-TCGGAGGGGCTAAAGCTGGTGT R-TGTCACACATGCGTGGGCGTT	233	(Dube et al. 2019)
Glutathione-S-transferase-pi	GST	Conjugation of electrophiles	F-ATTTTGGGACGGGCTGACA R-CCTGGTGCTCTGCTCCAGTT	81	(Salaberria et al. 2009)
Heat shock 70-kDa protein	HSP70	Protein chaperone	F-ACCACACCCAGTTATGTCGCCT R-CTTCCGCCCTATCAGCCGC	123	(Dube et al. 2019)
DNA Ligase	LIG	DNA repair	F-TGGTGCGATTTTGAAGTGTG R-GGTCCTGTGTCCTTGTGGTT	147	(Gagne et al. 2012)
Metallothionein	MT	Divalent metals homeostasis	F-GCTCTAAAACTGGCTCTTGC R-GTCTAGGCTCAAGATGGTAC	236	(Gagne et al. 2012)
Proliferating cell nuclear antigen	PCNA	Cell proliferation	F-CAGAGGACAACGCAGACACA R-CACGGCAGATACGGGCAAAC	156	(Gagne et al. 2013)
Superoxide dismutase (Cu/Zn cytosolic)	SOD	Oxygen radical scavenger	F-TGGTCCTGTGAAGCTGATTG R-TTGTCAGCTCCTGCAGTCAC	201	(Fontagne et al. 2008)
Secreted acidic cysteine rich glycoprotein	SPARC	Collagen and bone formation	F-TCACCCTGTACGAGCGCGATGA R-AGCTGACCGAACTGCCAGTGGA	187	(Dube et al. 2019)
Signal transducer & activator of transcription 3	STAT3	Response to cytokines, immunity	F-TGAGCCCTAAGCCTCCTGTT R-TCCCACTGATGTCCTTTTCCAC	99	In house
Ubiquitin	UB	Protein tag for degradation	F-CTACGCTCGCCTCCATCCT R-CCACAAAAAGCACCAAGCCAA	96	In house
Reference genes					
Elongation factor I α	EF1α	Aminoacyl tRNA transfer to ribosome	F-GAATCGGCTATGCCTGGTGAC R-GGATGATGACCTGAGCGGTG	141	(Bower and Johnston, 2009)
Hypoxanthine phosphoribosyl transferase I	HPRT	Purines salvage pathway	F-CCGCCTCAAGAGCTACTGTAAT R-GTCTGGAACCTCAAACCCTATG	255	(Bower and Johnston, 2009)
Prolylpeptidyl isomerase I	PPIA	Protein folding of new proteins	F-CATCCCAGGTTTCATGTGC R-CCGTTCAGCCAGTCAGTGTT	203	(Bower and Johnston, 2009)

The qPCR analyses were performed using SsoFast EvaGreen Supermix (Bio-Rad, Mississauga, ON, Canada) and CFX96 Touch Real-Time PCR Detection System (Bio-Rad). For each selected primer pair, a calibration curve (starting cDNA concentration: 2.5 ng, six serial dilutions, 2-fold) was established to obtain PCR efficiency (values between 90 and 110%) and limit of detection. Each reaction was run in duplicate and consisted of 5 μL cDNA (equivalent to 2.5 ng cDNA), 8 μL SsoFast EvaGreen Supermix (dNTPs, Sso7d-fusion polymerase, MgCl2, EvaGreen dye), 300 nM F- and R-primer, and DEPC-treated water (Thermo Fisher Scientific, Burlington, ON, Canada). Cycling parameters were 95 °C for 2 min, followed by 40 cycles of 95 °C for 5 s, 60 °C for 15 s. Amplification specificity was verified with a melting oC fr: 95 °C for 15 s, 57 °C for 5 s and slowly reaching 95 °C in 10 min. Each plate included a no-template control.

### Data analysis

Enzyme activity and other biomarker data were normalized with the total protein content of the homogenates while transcriptomic data were normalized by the reference genes. Normality and homogeneity of variances of the biomarker data were confirmed using the Shapiro-Wilk and Bartlett tests respectively. Difference between exposure concentration treatment groups of the REE mixture and controls was analyzed by one-way ANOVA and Tuckey’s post hoc test. The data were expressed as the mean with standard error for *n* = 9 individuals per treatment group. Data analysis was conducted with GraphPad Prism 8 (Version 8.4.0 (455) for Mac).

Data acquisition and analysis for gene expression data were performed with CFX Maestro (Bio-Rad, Mississauga, ON, Canada). Selected reference genes were elongation factor a (*ef1a*), hypoxanthine phosphoribosyl transferase (*hprt*), and prolylpeptidyl isomerase I (*ppia*). Genes were analyzed for their stability across the samples using the reference gene selection tool included in the CFX Maestro software (Bio-Rad). The data were normalized against housekeeping genes and expressed as fold changes of the control. Efficiencies obtained from the standard curves were entered manually to correct the Cq values. An ANOVA with a Tuckey’s post hoc test was performed on the gene expression data using CFX Maestro to account for significant changes between concentrations of the REE mixture. A Pearson’s correlation analysis was performed using GraphPad Prism 8 to determine significant relationships between gene expression data and biomarkers. Significance was set at *p* < 0.05 for all analyses.

## Results

### Acute toxicity of the REE mixture

The highest concentration tested solely for the determination of an LC_50_ was 100x. The LC50 value was 60X of the REEs mixture corresponding to the 18.2 mg/L Ce, 9.0 mg/L La, 7.5 mg/L Nd, 1.8 mg/L Pr, and 1.5 mg/L Sm (Table 3). The reported LC50 for the single REEs was included and revealed that Sm concentration in the mixture (1.36 mg/L) corresponded to the LC50 of Sm alone (1.6 mg/L) suggesting that toxicity was mainly samarium-driven in this mixture. The LC50 value of Pr alone (5.48 mg/L) was close to the Pr levels in the mixture (1.68 mg/L) suggesting some contribution of this element.

**Table 3:** Reported LC50 values of the selected REEs

REE	LC50 individual (mg/L)	LC50 values of mixture (60X)
La	120^a^	8.4
Ce	95 ^a^	7.8
Nd	60 ^a^	7.2
Sm	1.6	1.36
Pr	5.48^b^	1.68
		Total: 26.44 mg/L

^a^From Dubé et al. (2019), obtained from the acute/genomic threshold ratio. b. From the linear relationship between Hydra and rainbow trout (Blaise et al. 2018)

**Fig. 1:**
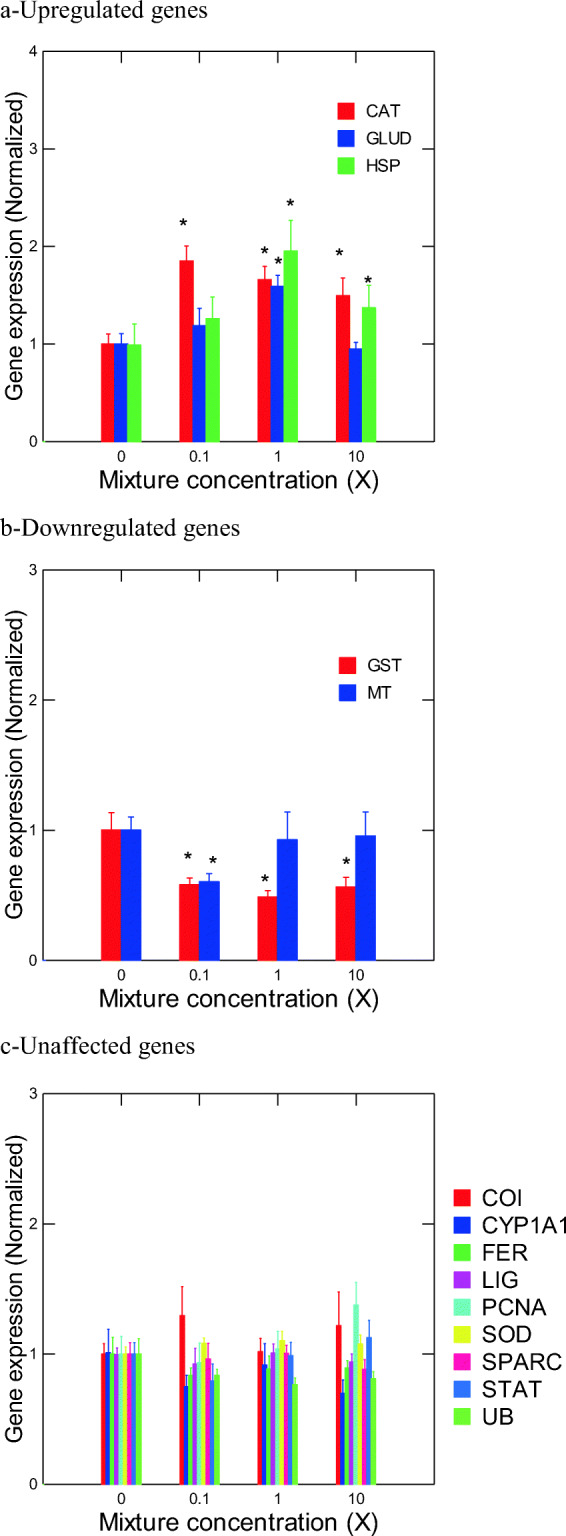
Gene expression changes induced by exposure to a REE mixture in rainbow trout liver. **a** Genes that were significantly upregulated in one or more concentrations of the mixture included CAT, GLUD, and HSP70. **b** Genes that were significantly downregulated in one or more concentrations of the mixture included GST and MT. **c** Genes without significant gene expression changes in any of the concentrations tested. The data represent the mean ± SEM. Asterisks (*) indicate a significant difference from the negative controls

We examined the expression of 14 genes of interest in rainbow trout liver samples (Fig. 1). We observed an upregulation of expression for all concentrations of the mixture for catalase (CAT), an enzyme protecting cells from oxidative stress (Fig. 1a). The genes for the 70 kDa heat shock protein (*hsp70*) and glutamate dehydrogenase (*glud*) were also upregulated at 10x concentration (Fig. 1a). Glutathione S-transferase (*gst*), an enzyme responsible for detoxification of electrophilic compounds, was downregulated (Fig. 1b). Metallothionein (*mt*), a protein involved in divalent metal detoxification, was also downregulated in fish exposed to the 1X concentration (Fig. 1b). The remaining nine genes of interest were not significantly changed in the mixture at the concentrations used (Fig. 1c). Correlation analysis between the gene expression data revealed that the following pairs were strongly correlated (*r* > 0.5; *p* < 0.001): *ub* and *fe*; *stat3* and *pcna*; *stat3* and *lig*; *hsp-lig*; *hsp-glud*; and *hsp-gst*. The influence of these genes was examined based on the number of correlation with other biomarkers: *glud* (8) = *hsp70* (8) < *cat* (7) = *lig* (7) = labile Zn < *sparc* (6) < others. If we consider the gene transcripts that were significantly changed by the REEs with influence potential, we obtain the following: *cat*, *hsp70*, and labile Zn. Hence, exposure to REEs leads to changes in reactive oxygen handling (*cat*), protein denaturation, and altered ammonium/Zn metabolism. Because of the strong correlations with some of these endpoints, we cannot exclude effects on *lig* and *gst*.

### Biomarker responses

**Fig. 2:**
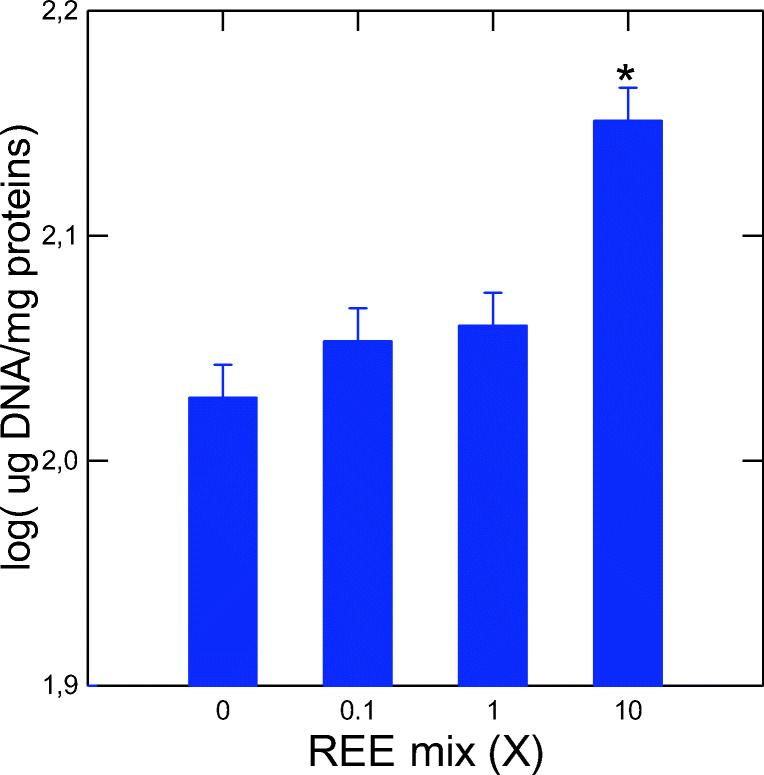
Evaluation of DNA strand breaks after 96 h exposure to a REE mixture in rainbow trout gills with the alkaline precipitation assay. Histograms show standard mean ± SEM. Significance was set at *p* < 0.05

**Fig. 3:**
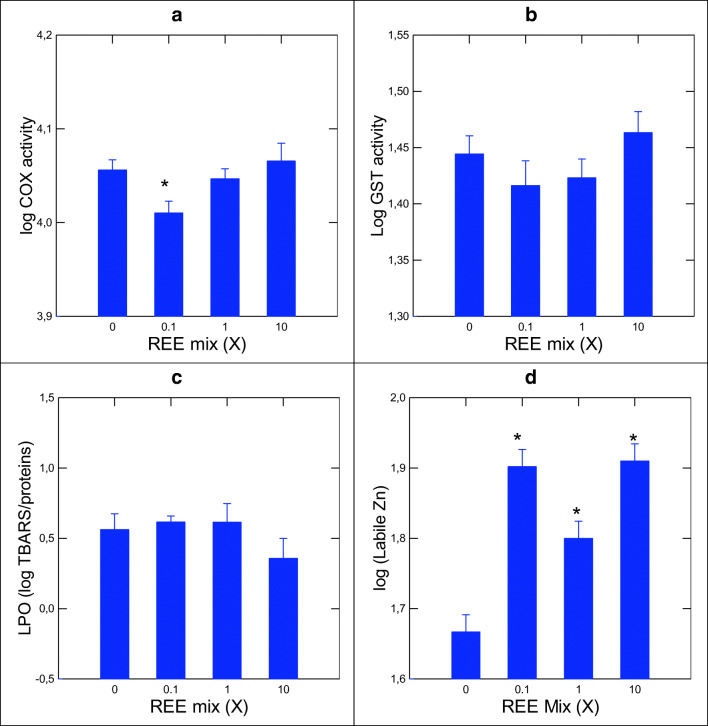
Evaluation of changes in enzyme activities and labile zinc levels in rainbow trout gills after 96 h exposure to a REE mixture. Histograms show standard mean ± SEM. The units for labile Zn were ng Zn/mg proteins). Significance was set at *p* < 0.05

DNA damage was measured using the alkaline precipitation assay in fish exposed to the REE mix (Fig. 2). The assay revealed DNA strand breaks at the highest concentration tested (10X). Electrophile conjugation (GST), inflammation (COX), oxidative stress (LPO), and intracellular labile zinc levels were analyzed after exposure to the REE mixture (Fig. 3a–d). COX activity showed a significant increase at a concentration threshold of 0.32X as determined by the Tuckey’s difference multiple comparisons test (Fig. 3a; COX *p* = 0.03). GST activity and LPO were not significantly affects by the REEs (Fig. 3b and c). Labile Zn levels were readily increased for all concentrations (< 0.1X). Correlation analysis revealed that the biochemical biomarkers were correlated between COX and GST activities (*r* = 0.68) (Table 4). Other strong correlations (i.e., *r* > 0.5; *p* < 0.01) were found for labile Zn and *sparc* (*r* = 0.51), and *sod* (*r* = 0.55) and *mt* gene expression in the liver (*r* = −0.31; 0.1 < *p* < 0.05). DNA strand breaks were, however, significantly correlated with GST (*r* = 0.48) and COX (*r* = 0.42) activities suggesting the involvement of oxidative stress.

**Table 4:**
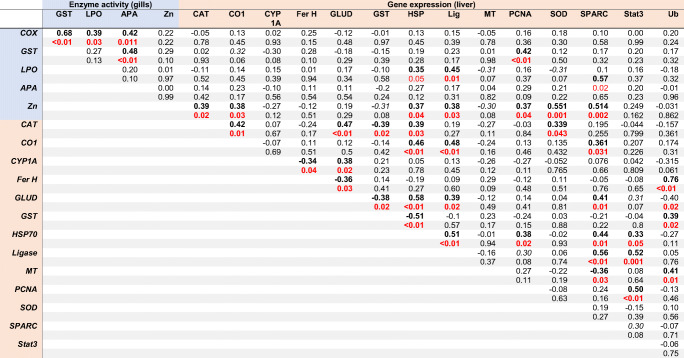
Correlation analysis of biomarker and gene expression data. Pearson’s *r* (white) and *p* value (gray) are given for each pair. *p* values of significant correlations (*p* < 0.05) are highlighted in red. That CAT was significantly correlated with CO1 (*r* = 0.48), GLUD (*r* = 0.47), GST (*r* = − 0.39), HSP70 (*r* = 0.39), and SOD (*r* = 0.34). Mitochondrial CO1 was significantly correlated with

**Fig. 4:**
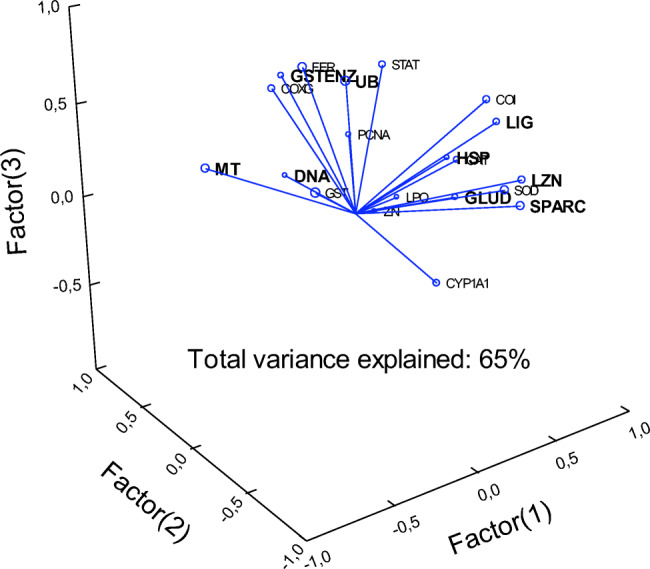
Principal components of biomarker data. The total explained variance was 65% by 3 components (axes). Biomarkers with the factorial weights > 0.7 are highlighted in bold

Principal component analysis was also performed to determine the biomarkers with the highest factorial weights (explaining the variance) and relationships between biomarkers (Fig. 4). The total variance was explained by 65% with 3 factors. The most important biomarkers (factorial weights > 0.7 in one of the 3 factors) were GST activity, DNA damage, *hsp70*, *sparc*, *ub*, *lig*, labile Zn, *mt*, and *glud*. The most important biomarkers were distributed in 3 distinct clusters or groups. The first cluster consisted in the *lig, sparc, glud*, labile Zn, and *hsp70*. The second cluster consisted in GST activity and *ub*. The third cluster involved *mt* and DNA damage. This suggests that these changes were able to explain most of the variance (65%) and includes the biomarkers found during above analysis in respect to the endpoints showing the highest number of correlations and changes with the exposure concentration. *Cat* was identified as one of the mostly correlated response biomarker and showed a factorial weight close to 0.7 at 0.68 and was closely associated with *hsp70*.

## Discussion

The adverse effects of REE on aquatic and terrestrial organisms were studied mostly in organisms exposed to single elements and many studies involved La and Ce only (Gonzalez et al. 2014). Previous studies on the toxicity and mechanisms of action of these elements revealed that each REEs might have distinct effects, complicating the identification of a universal mode of toxicity for this family. It is noteworthy that gene expression was determined in the liver while the biochemical biomarkers were done in gills tissues to satisfy tissue mass requirements to perform all the assays. Hence, this could introduce tissue discrepancy variations in the observed responses. The electronegativity and ionic radius (those close to calcium) were able to predict loss of survival in juvenile rainbow trout and Hydra (Dubé et al. 2019; Blaise et al. 2018). Based on the threshold concentration producing effects at the molecular level and toxicity (LC50), sublethal (chronic) effects were found at concentrations 600 times below the toxicity values in the present study suggesting that effects are likely to occur at in contaminated lakes by mining activities. The 1X mix corresponded to the 5 most abundant elements based on lakes concentrations under the influence of mining activities, hence the levels were higher than in undisturbed environments (Beaubien 2015). The ratio between sublethal effects and toxicity was higher than those obtained with single REEs with a ratio of 120 (Dubé et al. 2019). In some cases, gene expression was increased at lower concentrations followed by a decrease in expression. This suggests that the responses occurred at low concentration and reached saturation and exhausting of responses at higher concentrations.

Based on the relative concentrations of the REE of the mixture, the concentration of Sm in the mixture was very close to the LC50 of Sm in trout juveniles (Table 3). In trout exposed to Sm alone, *cyp1A1*, DNA damage (*gadd45*), *gst*, *hsp70*, and *mt* were upregulated and *cat*, *glud* genes were downregulated. In the present study, *gst* and *mt* were downregulated while *cat*, *glud*, and *hsp70* were upregulated indicating that only *hsp70* gene expression agreed with the present study. This suggests that other effects from the other elements in the mixture were at play. Thus, *hsp70* and DNA repair genes were affected by all of the individual 7 REEs examined (Dubé et al. 2019), which is consistent with the effects observed with *hsp70*, *lig*, and DNA damage data in the present study. If this holds true, REE mixtures could denature proteins (*hsp70*) and damage DNA in juvenile trout. This was further corroborated with the significant correlation between *hsp70* gene expression and the LC50 in rainbow trout juveniles exposed to 7 REEs (Dubé et al. 2019). Rainbow trout eggs have been shown to be very sensitive to La with an LC_50_ of 0.02 mg/L La in a 28-day study (Birge et al. 1979) corresponding to about 0.15X of the REEs mixture in the present study. Although three out of the five REEs in our mixture (La, Ce, Nd) had very high LC_50_ values individually, the mixture of five REEs induced significant effects in a number of genes at concentrations below the environmental level. One possible factor that could have influenced toxicity at high concentrations (LC50 assessment) is in the precipitation of REEs by the presence of phosphates in the exposure medium. Phosphate levels were below the detection limit (< 0.05 mg/L) in aquarium water, hence unlikely to cause significant precipitation. However, in a previous study by the same laboratory, evidence of precipitation was sometimes observed at concentrations > 40 mg/L in the same aquarium water of the present study (Dubé et al. 2019). The actual concentration in water for Sm and Ce was 38% and 35% of the nominal (added) concentration respectively but at concentrations > 40 mg/L. At concentrations below 40 mg/L, the measured concentration was between 75 and 104% of the nominal concentrations. Since the observed effects occurred at concentrations well below the LC50 for Sm, no precipitation was expected and no evidence of precipitation was observed at concentrations below 60X.

In the present study, the mixture of five REEs seemed to induce oxidative stress in the liver even below environmental levels (< 1X). While LPO in gills and *sod* expression in the liver remained unaffected, we observed a significant hepatic upregulation of *cat* and downregulation in *gst* and *mt* expressions for the selected concentrations of the mixture. In mice fed La, Ce, or Nd, oxidative stress was observed in the liver as demonstrated changes in the activities of SOD, CAT, glutathione peroxidase, glutathione (GSH), and LPO (Huang et al. 2011). Zebra mussels have shown antioxidant or prooxidant activity depending on La concentrations (Hanana et al. 2017). Although some evidence of oxidative stress was shown, other studies demonstrated, however, opposite antioxidant effects (Pagano et al. 2015). REEs have displayed hormetic effects related to oxidative status and antioxidant capacities on multiple occasions (Gonzalez et al. 2014). Based on the lack of LPO, our data support the notion that the REEs mixture did not produce oxidative damage. This suggests that the REE mixture displayed no antioxidant effects. However, the increase of labile Zn in the gills with the absence of *mt* gene expression in the liver suggests that the REEs were able to increase the labile fraction of Zn and sparc expression (cysteine-rich osteonectins) in cells perhaps through REE displacement of bound Zn to proteins such as osteonectins and Cu, Zn–SOD. Indeed, the levels of labile Zn were strongly correlated with *sod* and *sparc* expressions suggesting involvement of antioxidant responses and interaction with collagen and Ca fixation in bones. More research will be needed to examine the interaction of REE to osteonectins. It was shown that La could bind to collagen mimicking peptides (Sun et al. 2019) which are related to *sparc* gene expression. However, it is not known if the other REEs, especially Sm, could bind collagen and produce changes in *sparc* gene expression. Other REEs ions were shown to bind collagen peptides forming helical nanoropes (He et al. 2016). The lanthanides that promoted the winding of collagen peptide into nanoropes were La, Eu, and Tb. Recent evidence showed that gadolinium, La, and terbium could promote bone formation as they serve as templates to initiate osteogenesis in mammalian organisms (Liao et al. 2019; Hu et al. 2018; Liu et al. 2014). However, this has yet to be observed directly in aquatic organisms and the present data suggests that similar effects could be observed in fish. *Sparc* codes for osteonectin, a high affinity collagen binding glycoprotein that fixes calcium, initiate mineralization and promotes crystal formation. *Sparc* gene expression was one the most influential responses according to principal component analysis, strength and number of correlations in the present study. The impacts of REEs on bone/shell growth should be examined more closely in future work.

Genes involved in detoxification and oxidative stress (*mt* and *GST*) was downregulated at low concentrations of the mixture. The downregulation of MT gene expression was consistent with the increase in labile Zn but was only marginally correlated (*r* = − 0.30; *p* = 0.08). In zebra mussels exposed to Sm, an upregulation of *gst* gene expression was observed at > 10 μg/L (Hanana et al. 2018), while La decreased GST activity at the highest concentration tested (1250 μg/L) after 14 days (Hanana et al. 2017). MT levels were not affected by La in the exposure conditions chosen (Hanana et al. 2017) but were induced in juvenile fish exposed to Sm (Dubé et al. 2019). *Mt* gene expression was not correlated with any of the other end points with the exception with SPARC gene expression (*r* = − 0.36). The relationship between them, if any, is not clear. Both encode for cysteine containing proteins involving perhaps the binding of Ca ions (Sage et al. 1989) and perhaps Cu^2+^ or Zn^2+^ as with MT. The extracellular matrix contains also various metalloproteases (Zn?) which are involved in the de-gelation of the extracellular matrix promoting permeation by (cancerous) cells in tissues (Velasco et al. 2000). A double role for MT, one of protection and promotion, in carcinogenesis, has been proposed (Rodrigo et al. 2020). This suggests that REEs could disrupt cell differentiation and proliferation processes.

Exposure of rainbow trout juvenile lead to the formation of DNA strand breaks although no increase in *lig* gene expression was observed. The genotoxic potential was previously reported in trout exposed to Sm, gadolinium, cerium, La, erbium, and neodymium based on *gadd45* (growth arrested DNA repair activity) gene expression (Dubé et al. 2019). The expression of *gadd45* was expressed at a threshold concentration of 0.12 mg/L corresponding to a 5X concentration in the REEs mixture based on Sm. Sm significantly decreased strand breaks at 0.25 mg/L (corresponding to 10X concentration of the REEs mixture) in mussels compared to controls suggesting decreased repair activity (Hanana et al. 2018). The same calculation was made for La (8X), Ce (13X), Nd (5X). Based on the composition of the REEs mixture, Sm, Nd, and La could explain the levels in DNA strand breaks observed at 10X. Genotoxicity has been previously observed for Ce but not La such as mitotic aberrations in sea urchin embryos at 1.4 mg/L corresponding to the 5X concentration of the mixture (Oral et al. 2010). Micronuclei and chromosomal aberrations in bone marrow of mice exposed to Pr(III) and Nd(III) in intraperitoneal injected mice (Jha and Singh 1995). However, La did not display genotoxic effects in zebra mussel when assessing DNA strand breaks up to concentrations of 1.25 mg/L (Hanana et al. 2017). Based on these studies, the following REEs would be the most suspect in respect to genotoxicity: Sm, Nd, and Ce.

## Conclusions

The data revealed that the effects of REEs on fish survival could be explained by Sm with no evidence of synergitic or antoganist effects in rainbow trout juveniles. Gene expression and biochemical markers revealed that sublethal effects could be shown at concentration 600 times less than the acute lethality. This corresponds to concentrations of REEs mixture below 1X suggesting the possibility of toxic effects in the environment especially those contaminated by mining activities. The major sublethal effects involved the following pathways: DNA strand breaks (genotoxicity), labile Zn, *cat*, *gst*, *hsp70*, *sparc*, *mt*, and *glud* as determined by principal component analysis and concentration-dependent effects. The results suggest that REE mixtures and these effects are likely to occur in environments under the influence of mining activities.

## Data Availability

The data are available upon demand by request to the corresponding author.
